# Undersurface rotator cuff repair for partial-thickness tears using the Opus knotless system: a prospective cohort of 1000 consecutive cases with a 1.4% retear rate

**DOI:** 10.1016/j.jseint.2026.101778

**Published:** 2026-08-07

**Authors:** Victor Chen, Dineth Fernando, Alvin Deng, Chen Zhang, Stone Sima, James Bilbrough, Eric Changbean Kang, Ala Hawa, Mina Shenouda, Lisa Hackett, Patrick Lam, George A.C. Murrell

**Affiliations:** a Orthopaedic Research Institute, St George Hospital Campus, University of New South Wales, Sydney, Australia; b Orthopedics Division, Department of Surgery, Faculty of Medicine, Yarmouk University, Irbid, Jordan

**Keywords:** Rotator cuff repair, Partial thickness, Retear, Range of motion, Pain, Outcomes

## Abstract

**Background:**

Partial-thickness rotator cuff tears are a common cause of shoulder pain and disability. There are few large prospective cohort studies evaluating outcomes following arthroscopic repair, and reported structural failure rates vary widely across techniques. The purpose of this study was to (1) evaluate the structural and clinical outcomes of an arthroscopic undersurface knotless inverted mattress repair for partial-thickness rotator cuff tears, (2) assess the effect of surgical experience on outcomes, and (3) place these outcomes in the context of published series of alternative techniques.

**Methods:**

A prospectively collected cohort of 1000 consecutive patients with articular- or bursal-sided partial-thickness supraspinatus tears >50% thickness underwent arthroscopic repair using the Opus knotless system (Smith & Nephew) with an undersurface knotless inverted mattress technique. Structural integrity was assessed using ultrasound at a minimum of 6 months. Pain, range of motion, and strength were assessed pre-operatively and at 6 weeks, 12 weeks, and 6 months.

**Results:**

The cohort comprised 570 males and 430 females with a mean (±standard deviation) age of 56 ± 11 years (range, 18-87). Tear area was 1.5 ± 1.0 cm^2^, and median anchor number was 1 (range, 1-5). Repairs were intact in 986 patients at 6 months, with a retear rate of 1.4% (95% confidence interval: 0.8-2.3%). Seven patients (0.7%) underwent revision rotator cuff repair, and no patients required reoperation for infection or post-operative stiffness. Significant improvements were observed across pain, range of motion, strength, and functional outcomes (*P* < .01). A learning curve was identified, with retear rates peaking at 5% between cases 100-200 and declining to ≤1% after approximately 200 cases.

**Conclusion:**

Arthroscopic undersurface knotless inverted mattress repair of partial-thickness rotator cuff tears achieved a retear rate of 1.4%, with no reoperations for infection or post-operative stiffness and consistent improvements across all clinical outcome measures. Retear rates declined after approximately 200 cases, a pattern consistent with a learning-curve effect, although this descriptive moving-average analysis is exploratory and was not subjected to formal change-point modeling. The favorable structural outcomes and short operative time observed with this technique may relate, in part, to minimal disruption of the remaining tendon and subacromial bursa and the direct intra-articular visualization afforded by this undersurface approach.

Rotator cuff tears are a common cause of shoulder pain. The prevalence of cuff tears increases with age and therefore represents a significant cause of morbidity within aging populations.^8^ While partial-thickness tears are often managed non-operatively, many progress over time, and tear enlargement is reported in 44% of patients within 5 years of diagnosis, accompanied by increasing pain and declining shoulder function.^19^ Symptomatic partial thickness rotator cuff tears often present with complaints of night pain, pain with overhead activities, decreased throwing strength, and deep posterior shoulder pain accompanied by fatigue of surrounding muscles.^10^

Rotator cuff repair techniques have evolved from open procedures to mini-open approaches and, more recently, to fully arthroscopic methods.^39^^,^^41^ Surgical management of partial-thickness rotator cuff tears includes arthroscopic débridement, *in situ* repair, and tear completion followed by repair, and more recently augmentation with a biological patch, using a range of suture configurations and arthroscopic visualization methods.^17^^,^^26^^,^^29^^,^^32^ Biological onlay grafts involve placement of a patch on top of a tendon repair construct, in which an exposed area is covered to, in theory, provide strength and a scaffold for tendon growth.

We developed an undersurface rotator cuff technique which involves converting a partial-thickness tear into a small full-thickness tear under direct vision, deploying inverted mattress sutures directly into the torn tendon, and then placing and tensioning knotless anchors to the insertion site while maintaining the arthroscope within the glenohumeral joint.^9^^,^^31^ The outcomes of this technique in partial-thickness tears are undetermined. It is also unclear how the results of this technique compare with other published techniques of surgically managing partial-thickness rotator cuff tears. The aim of this study, therefore, was to evaluate the effectiveness of this arthroscopic technique for the repair of partial-thickness rotator cuff tears and place these outcomes in the context of published series of alternative techniques. We hypothesized that the undersurface knotless inverted mattress technique would achieve a low rate of structural retear and significant improvements in pain, range of motion, and strength, and that retear rates would be numerically comparable to or lower than those reported for alternative arthroscopic techniques.

## Methods

This was a prospective single-surgeon cohort study (Level of evidence 4) analyzing prospectively collected data from consecutive patients who underwent primary arthroscopic rotator cuff repair using an undersurface knotless inverted mattress suture technique.

The ethics approval of this study was granted by the South-Eastern Sydney Local Health District Human Research Ethics Committee (2019/ETH14049).

### Inclusion and exclusion criteria

Patients with an articular, intrasubstance, or bursal-sided partial-thickness supraspinatus tear greater than 50% of tendon thickness who underwent primary arthroscopic rotator cuff repair by a single surgeon (G.A.C.M.) using an undersurface knotless suture inverted mattress technique between March 2005 and September 2024 were eligible for inclusion. The operative indication was a symptomatic partial-thickness supraspinatus tear measuring at least 50% of tendon thickness in a patient who elected surgical management. Pre-operative tear characterization was performed by specialist musculoskeletal ultrasound. We have found ultrasound to be highly accurate for measuring tear depth, thickness, and location (articular surface, intrasubstance, or bursal side) when performed by an experienced musculoskeletal sonographer, with 97% sensitivity and 100% specificity for tears ≥50% tendon thickness.^2^^,^^7^^,^^24^

Patients were excluded if they had: 1) previous rotator cuff repairs, 2) incomplete repairs, 3) irreparable tears, 4) associated fractures, or 5) severe glenohumeral arthritis.

### Surgical technique

**Figure 1:**
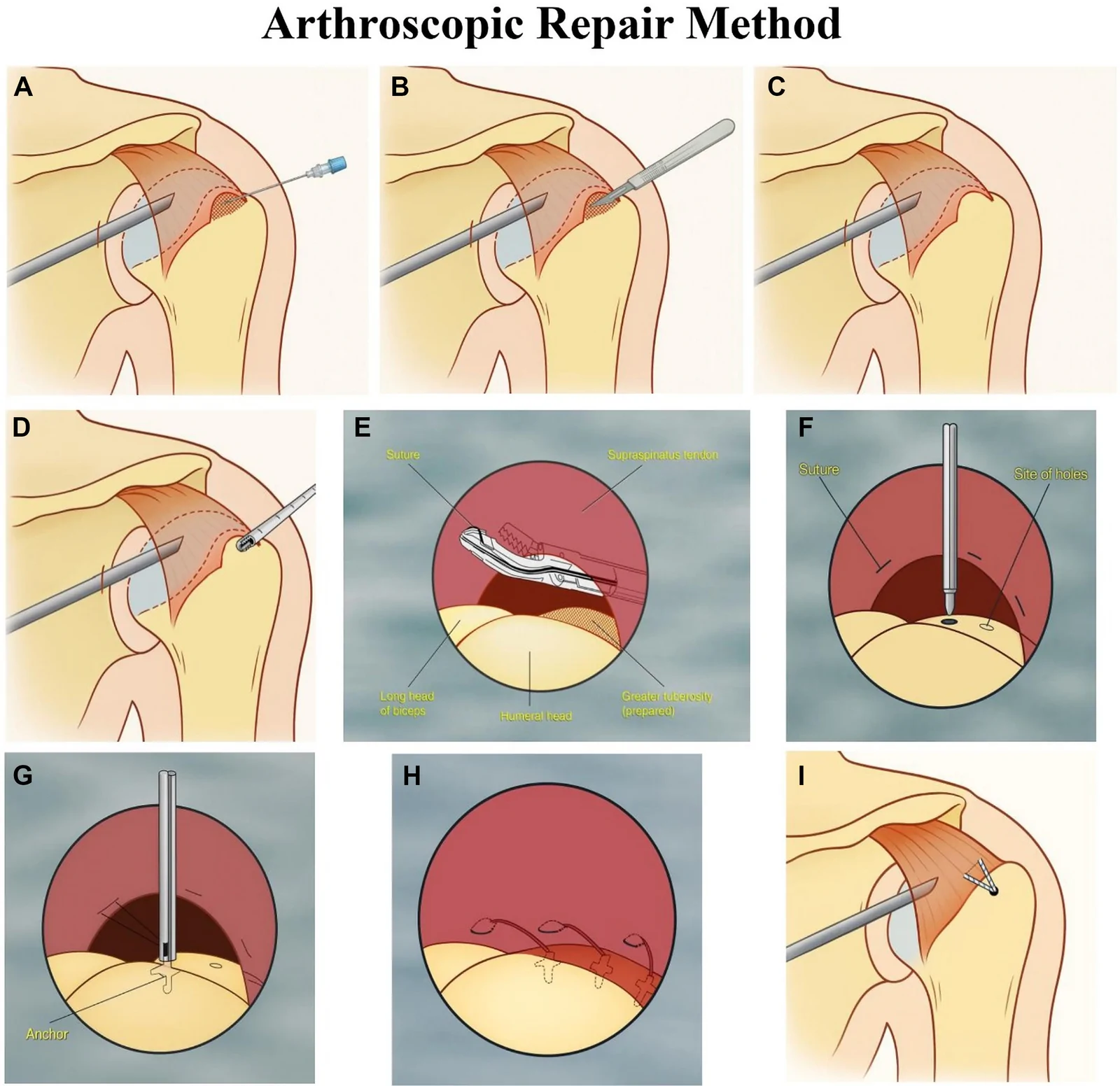
Undersurface approach to arthroscopic rotator cuff repair. (**A**) Torn tendon is visualized from the undersurface with the arthroscope and the torn tendon is located with a spinal needle. (**B**) Partial-thickness tear is converted to a full-thickness tear with a scalpel blade. (**C**) Full thickness torn tendon edge is visualized from the glenohumeral joint. (**D**) Landing site on the greater tuberosity is prepared with an arthroscopic shaver. (**E**) An Opus SmartStitch device is used to pass sutures through the torn edge of the tendon while being visualized from an undersurface position. (**F**) A T-handle punch is used to prepare tunnels for suture anchors. (**G**) Suture ends were prepared with the Opus Magnum 2 suture anchor and placed into T-handle tunnel sites. (**H**) Tension lock mechanism was used to deploy the anchor and tension the repaired tendon. (**I**) Completed single-row tension band rotator cuff repair.

A complete surgical technique is described in Video 1. Patients were placed into a beach chair position with interscalene block and sedation for anesthesia. A posterior port was made 2 cm medial and 2 cm inferior to the posterior-lateral corner of the acromion for insertion of the arthroscope into the glenohumeral joint. Through the posterior portal, an initial diagnostic arthroscopy was performed to visually confirm the rotator cuff tear (Fig. 1). The location of the lateral portal was determined by passing a spinal needle through the torn tendon. The location of the lateral port is ideally midway between the anterior and posterior edges of the torn tendon and parallel or slightly superior to the rotator cuff landing site. This position of the lateral portal allows for unobstructed access for landing site débridement and rotator cuff repair. Furthermore, this portal position provides optimal access for anchor insertion into the greater tuberosity. Arthroscopic cannulas are not utilized.

To begin the repair, partial-thickness tears were converted to a full-thickness tear with a No. 11 blade inserted through the lateral port after localization with a spinal needle. The landing site of the tendon was débrided with a 4.5 mm arthroscopic shaver, which induced bleeding to enhance the healing potential of the tendon-bone interface. An Opus SmartStitch device (Smith & Nephew, Andover, MA, USA) was used to pass sutures through the torn edge of the tendon via the lateral portal in an inverted mattress configuration. A T-handled punch was inserted through the lateral portal to make the tunnel for the suture anchors. The location of the tunnel was ideally located in the lateral portion of the landing site. Suture ends from the tendon were passed through an Opus Magnum 2 suture anchor (Smith & Nephew, Andover, MA, USA), and the anchors were then placed into the T-handle tunnel sites. The tension lock mechanism on the Opus Magnum 2 was used to tension the sutures and deploy the anchor. Acromioplasty was not performed, bursectomy was not performed, and the clavicle and biceps were left alone.

### Rehabilitation

Rehabilitation consisted of pre-operative and post-operative sessions. Patients attended a group education session with the physical therapist a week prior to surgery. Post-operatively, patients were placed in an Ultrasling with a 30° abduction pillow (DJO, Frenchs Forest DC, NSW, Australia) for 6 weeks. Rehabilitation in the first 6 weeks consisted of gentle passive range of motion exercise. At 6 weeks post-operatively, each patient underwent review by a physical therapist and was then initiated on isometric strengthening exercises. Overhead activity outside of these exercises was discouraged. Patients were transitioned to active resistance exercises at 3 months following review by the same physical therapist. Patients were also permitted limited overhead activity of less than 15 minutes duration. Lifting restrictions varied from 2 to 5 kg dependent on individual patient progress. Unrestricted shoulder function was permitted at 6 months post-operatively after review with ultrasound.

### Clinical outcomes

Clinical outcomes were assessed pre-operatively and at 6 weeks, 12 weeks, and 6 months. Shoulder pain and function were performed according to our previously published protocol.^30^ Overall shoulder function was assessed using a 5-point patient-rated Likert scale (very bad, bad, poor, fair, good) adopted from the response structure of the L'Insalata Shoulder Rating Questionnaire,^25^ an instrument shown to be valid, internally consistent (Cronbach α 0.71-0.90), reproducible (Spearman–Brown 0.94-0.98), and responsive to clinical change.^12^^,^^43^ Passive shoulder range of motion, including forward flexion, abduction, and internal rotation, was measured visually by trained examiners using validated protocols.^14^ Strength was measured with a hand-held dynamometer (HFG-45 Force Gauge) using validated protocols.^15^

### Repair integrity

Patients returned for assessment of repair integrity at 6 months. All post-operative ultrasound assessments were performed by 2 experienced specialist musculoskeletal sonographers using a standardized protocol whose accuracy and reliability have been established at our institution (97% sensitivity and 100% specificity for tears ≥50% thickness).^2^^,^^7^^,^^13^^,^^34^ Equivocal or thinned repairs were re-examined at 6 and 12 months; magnetic resonance imaging (MRI) confirmation was not used, as ultrasound by these sonographers has been shown to be at least as accurate as MRI in our setting.^3^ The sonographers were blinded to the clinical outcomes, operative findings, and case number at the time of post-operative imaging. Retear was defined as full-thickness loss of continuity of the repaired tendon on ultrasound.^7^^,^^24^

### Statistical analysis

Statistical analysis was performed using SPSS (IBM Corp., Armonk, NY, USA). Descriptive statistics were used to characterize the age and sex demographics. Repeat measures analysis of variance were used to compare range of motion and strength with post hoc pairwise comparisons made using Bonferroni-corrected *t*-tests. To guard against type I error from multiple comparisons, all post hoc pairwise comparisons were Bonferroni-corrected. All repeated measures analysis of variance were corrected using the Greenhouse-Geisser method where sphericity was violated as per Mauchly's test. Pain outcomes across follow-ups were compared using a Friedman test with post hoc pairwise comparisons made using Bonferroni-corrected Wilcoxon signed rank tests. Correlations between ordinal variables were assessed using Spearman rank correlation coefficients.

The dataset was ordered in ascending chronological order based on date of operation, and patients were assigned a corresponding case number from 1 to 1,000. Moving averages were plotted for operative time and retear rate by first calculating the means for the first 100 patients (case 1 – case 100), then continued by moving along 1 patient (case 2 – case 101) and recalculating new averages until the end of the dataset was reached (case 901 – case 1,000).

To provide context, retear rates from published series of alternative arthroscopic repair techniques were summarized. Proportions were logit-transformed and combined using the DerSimonian–Laird estimator, with between-study heterogeneity quantified using the I^2^ statistic. This was a contextual pooled summary rather than a formal systematic review or meta-analysis. Estimates were stratified by surgical technique (conversion repair and *in situ*/transtendon repair).

## Results

### Demographics

There were 1,000 consecutive patients with partial-thickness rotator cuff tears who underwent arthroscopic rotator cuff repair using the undersurface during the study period who met the inclusion criteria. Of these, 570 were male and 430 were female, with a mean age of 56 ± 11 years (range, 18-87). The mean tear area was 1.5 ± 1.0 cm^2^ (range, 0.1-9.0 cm^2^), and the median number of anchors used was 1 (range, 1-5).

### Repair integrity

**Figure 2:**
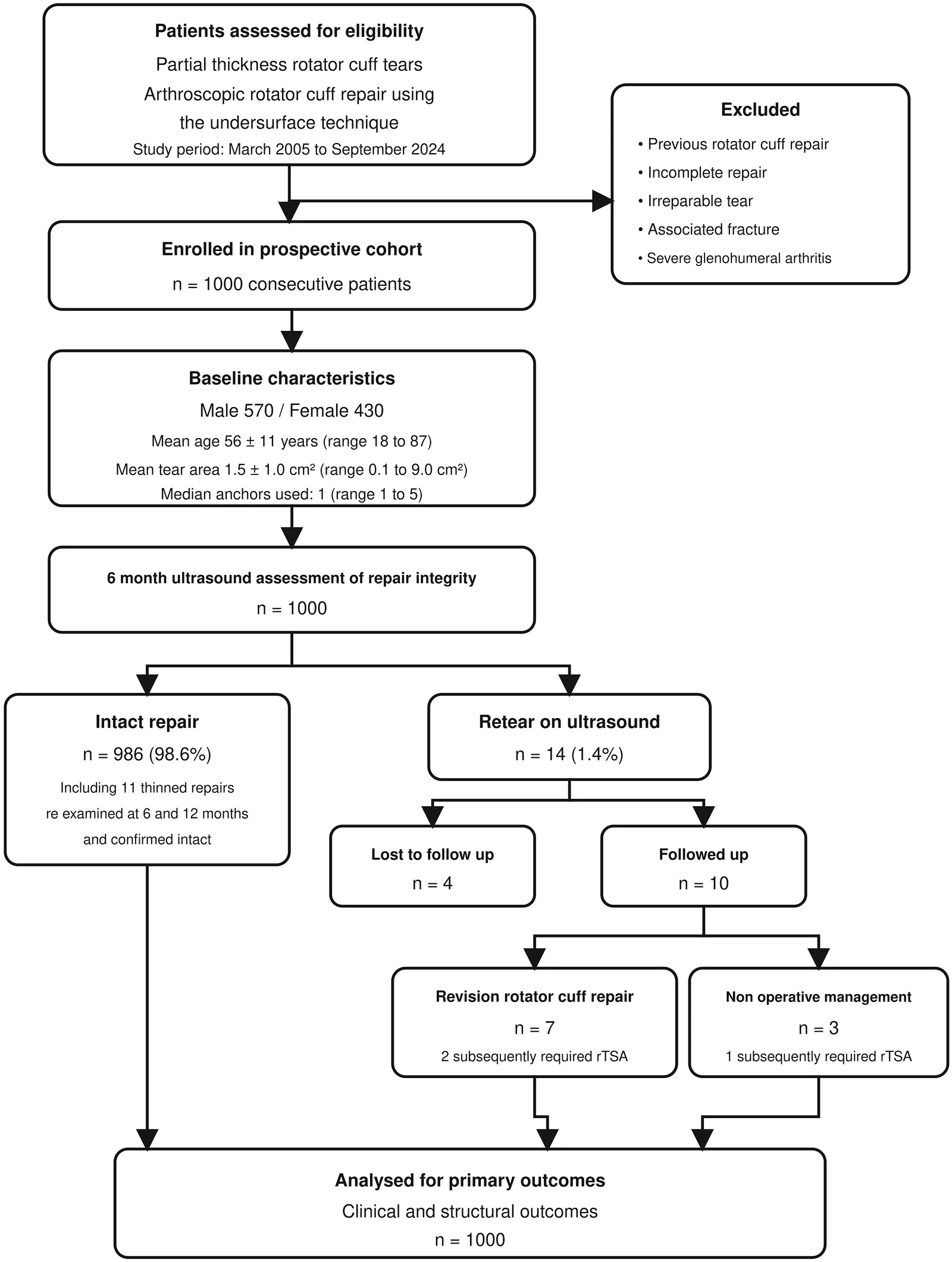
STrengthening the Reporting of OBservational studies in Epidemiology (STROBE) flow diagram for the study cohort. Patients eligible for inclusion had an articular- or bursal-sided partial-thickness supraspinatus tear >50% thickness and underwent primary arthroscopic undersurface repair by a single surgeon (G.A.C.M.) between March 2005 and September 2024. Of 1,000 consecutive enrolled patients, 986 (98.6%) had intact repairs and 14 (1.4%) had a retear on ultrasound at 6 months; of those with retears, 4 were lost to follow-up and 10 were followed up (7 underwent revision repair, of whom 2 progressed to reverse total shoulder arthroplasty; 3 were managed non-operatively, of whom 1 progressed to reverse total shoulder arthroplasty). *rTSA*, reverse total shoulder arthroplasty.

A total of 986 patients (99%) had intact repairs at 6 months (Table I). Eleven patients demonstrated thinned repairs that were re-examined at 6 and 12 months and noted to be intact. In total, 14 retears were detected on ultrasound examination at 6-month follow-up, resulting in an overall retear rate of 1.4% (Fig. 2). Of these 14 retears, 6 were from bursal-sided tears.

**Table I:** Patient and surgical characteristics according to repair integrity at 6 months.

Variable	Intact (n = 986)	Retear (n = 14)
Age (yr, ±SD)	55.7 ± 11.3	60.0 ± 13.5
Female, n (%)	427 (43.3%)	4 (28.6%)
Male, n (%)	559 (56.7%)	10 (71.4%)
Tear area (mm^2^ ±SD)	155.4 ± 217.8	152.8 ± 109.3
Anchor number (number ±SD)	1.5 ± 0.6	1.9 ± 0.9
Average case number (number ±SD)	502.7 ± 288.3	345.3 ± 292.3

*SD*, standard deviation.

Continuous variables are presented as mean ± standard deviation and sex as n (%). Tear area is reported in mm^2^. Case number represents the mean chronological case identifier.

**Figure 3:**
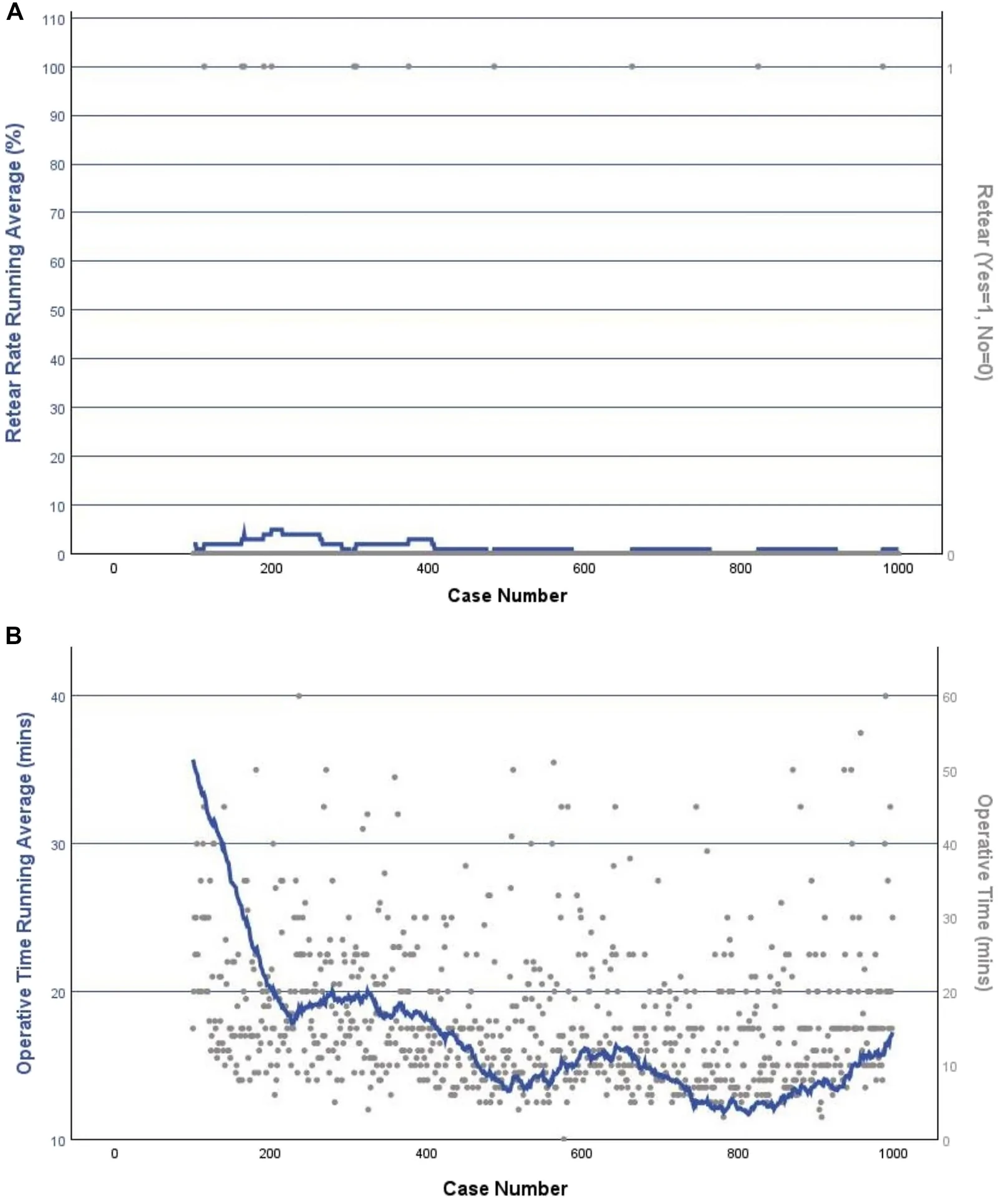
Rolling average analysis of (**A**) retear rate and (**B**) operative time over case number. Moving averages were plotted for operative time and retear rate by first calculating the means for the first 100 patients (case 1–case 100), then continued by moving along one patient (case 2–case 101) and recalculating new averages until the end of the dataset was reached (case 901–case 1,000). Gray point on the retear rate graph represent individual retear cases.

A moving average analysis of retear rate versus case number was performed to determine the effect of increasing surgeon experience. The moving average of the retear rate peaked at 5% between cases 100 and 200, and then declined and remained at ≤1% after approximately 200 (Fig. 3).

### Operative time

The median operative time (skin incision to suture placement) was 15 minutes (interquartile range [IQR] of 10-23 minutes; 95% confidence interval [CI]: 17.3-18.8 minutes) and ranged between 3 and 60 minutes. Operative time reduced with increasing case number (R^2^ = 0.162, *P* < .01). A moving average analysis of operative time versus case number was performed to determine the effect of increasing surgeon experience (Fig. 3).

### Revision surgery

No additional surgical procedures were required for infection or for post-operative stiffness in any patient in this cohort. Of the 14 patients with retears, 4 were lost to follow-up. Of the remaining 10 patients, 7 underwent revision rotator cuff repair, and 3 were managed non-operatively. Of the 7 patients who underwent revision repair, 2 subsequently required reverse total shoulder arthroplasty. Of the 3 patients managed non-operatively, 1 subsequently underwent reverse total shoulder arthroplasty.

### Outcomes

#### Patient range of motion, strength, and pain outcomes

**Figure 4:**
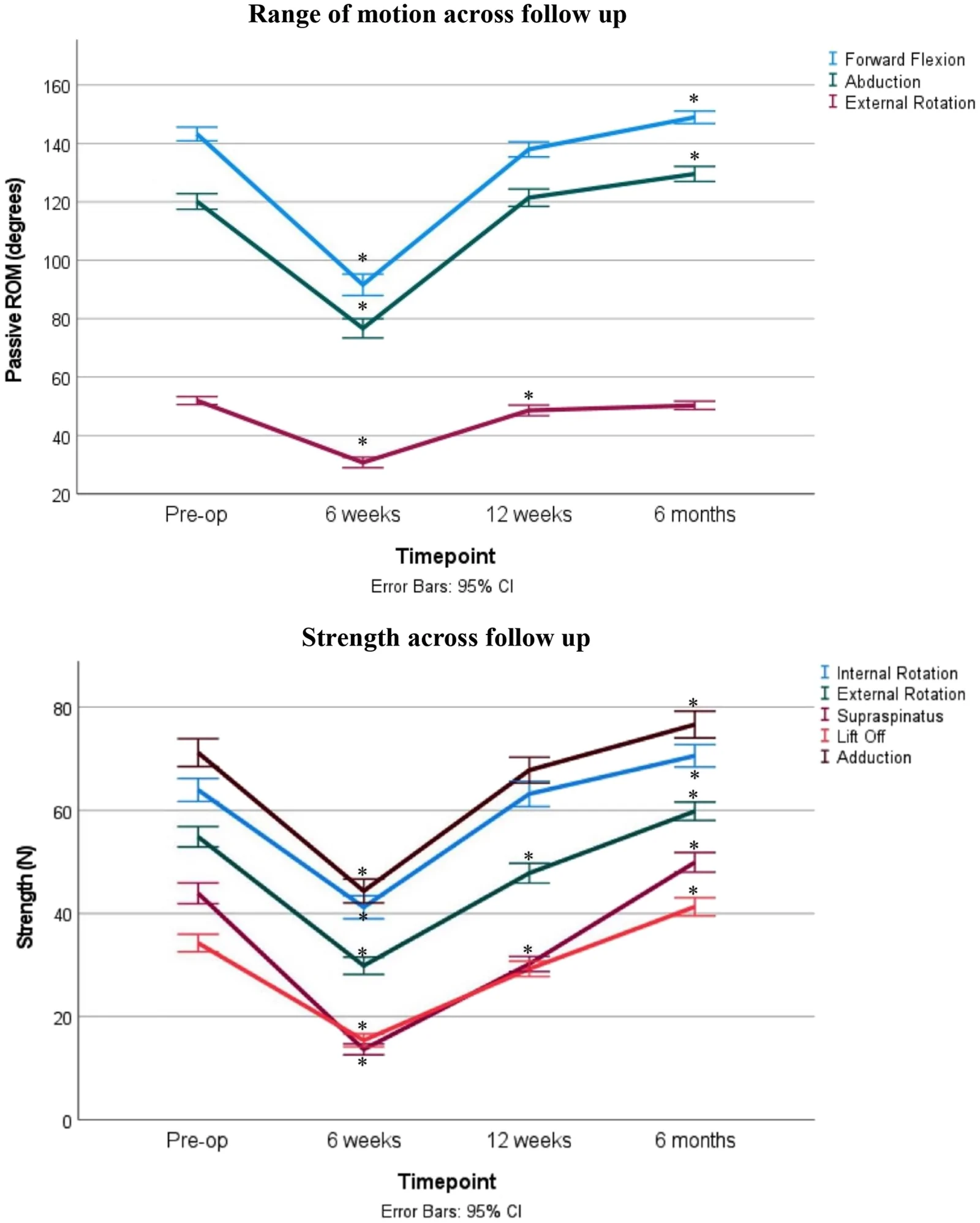
Patient range of motion and strength across the follow-up periods. ∗Indicates *P* < .05 when follow-up measurements are compared to pre-operative values. *CI*, confidence interval; *ROM,* range of motion.

All passive range of motion measurements had statistically significant changes across the 4 time points (pre-operative, 6 weeks, 12 weeks, and 6 months) (Fig. 4). Respective pre-operative range of motion values were correlated with 6-month post-operative values for forward flexion (ρ = 0.30, *P* < .01), abduction (ρ = 0.13, *P* < .01), and external rotation (ρ = 0.26, *P* < .01).

All strength measurements exhibited significant changes at each of the 4 time points (pre-operative, 6 weeks, 12 weeks, and 6 months) (Fig. 4). Respective pre-operative strength values were correlated with post-operative values for internal rotation (ρ = 0.57, *P* < .01), external rotation (ρ = 0.51, *P* < .01), supraspinatus strength (ρ = 0.40, *P* < .01), lift-off (ρ = 0.39, *P* < .01), and adduction (ρ = 0.53, *P* < .01) at 6 months follow-up.

**Figure 5:**
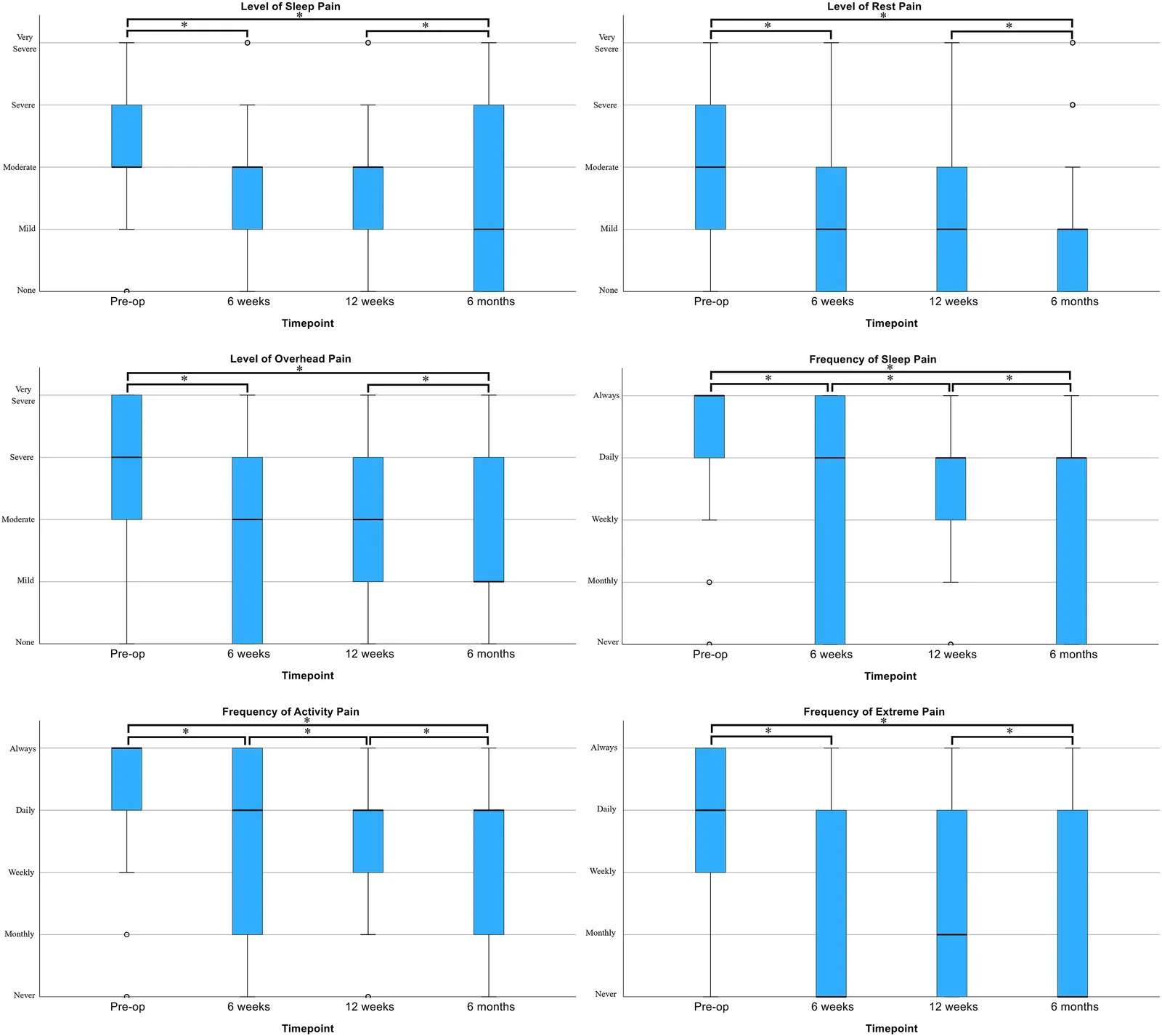
Patient-rated pain levels across follow-up period. Pain level was rated on a scale: none, mild, moderate, severe, and very severe. Pain frequency was rated on a scale: never, monthly, weekly, daily, and always. ∗Indicates *P* < .05.

All pain measurements exhibited significant improvements at each of the 4 time points (pre-operative, 6 weeks, 12 weeks, and 6 months) (Fig. 5). Pain levels at rest, during overhead activity, and during sleep significantly decreased between pre-operative to 6 weeks (*P* < .05). Pain in the overhead position, at rest, and during sleep also decreased between 12 weeks and 6 months (*P* < .05). Similarly, the frequency of extreme pain, pain during activity, and pain during sleep also declined significantly across time points (*P* < .05).

### Overall shoulder function

**Figure 6:**
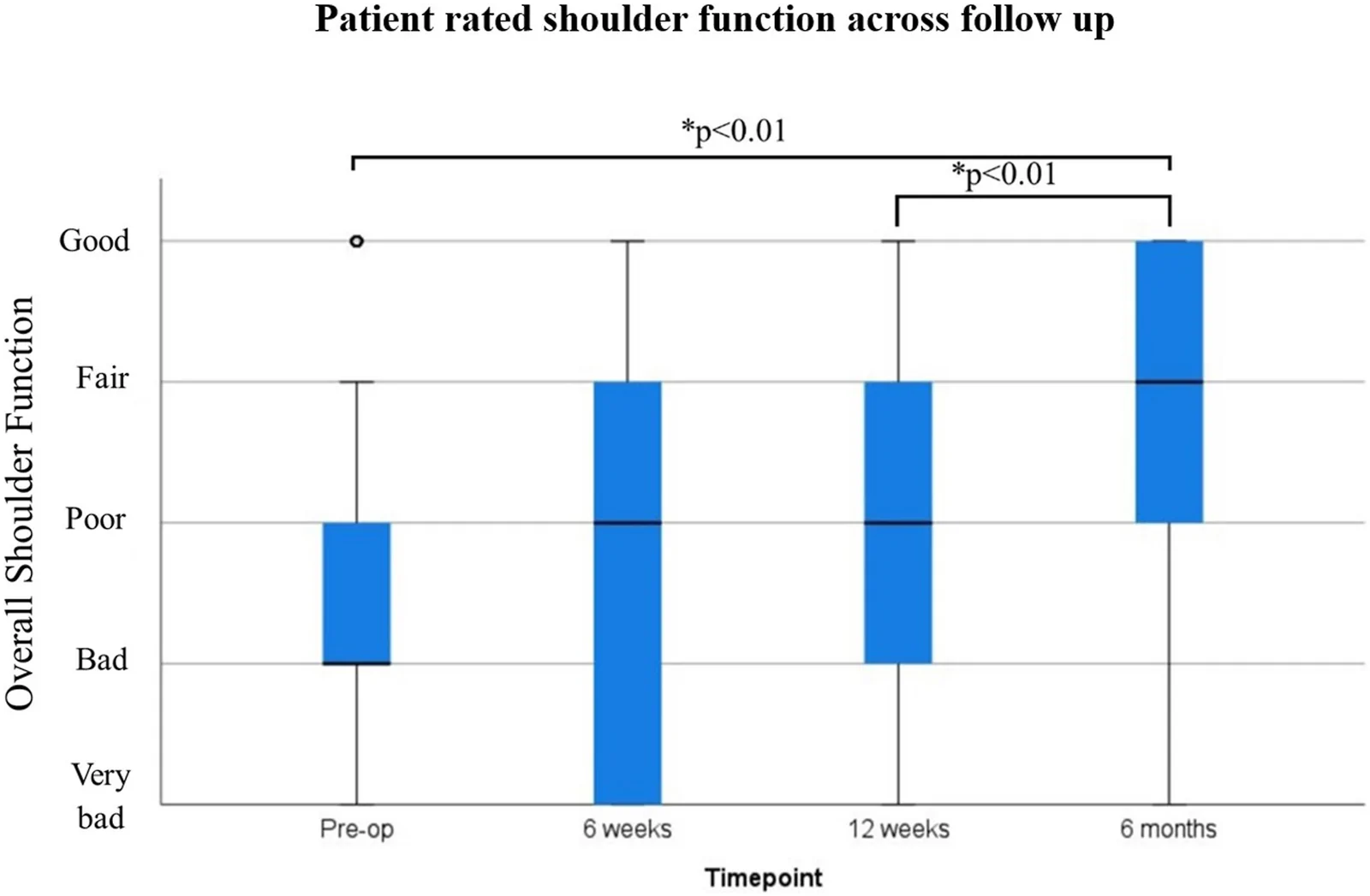
Patient-rated shoulder function across the follow-up period.

Subjective patient-reported rating of overall shoulder function (Fig. 6) demonstrated significant differences across the 4 time points per Friedman test analysis. Median rating pre-operatively was 1 (IQR: 1-2), corresponding to “bad.” This improved to a median rating of 2 (IQR: 1-3) at 6 weeks (z = 7.04, *P* < .01), corresponding to “poor.” Ratings improved from 12 weeks to 6 months (z = 10.77, *P* < .01) to a median of 3 (IQR: 2-4), corresponding to “fair” overall shoulder function. There was a statistically significant improvement in overall shoulder function rating from pre-operative to 6 months (z = 19.46, *P* < .01).

### Comparative studies

**Figure 7:**
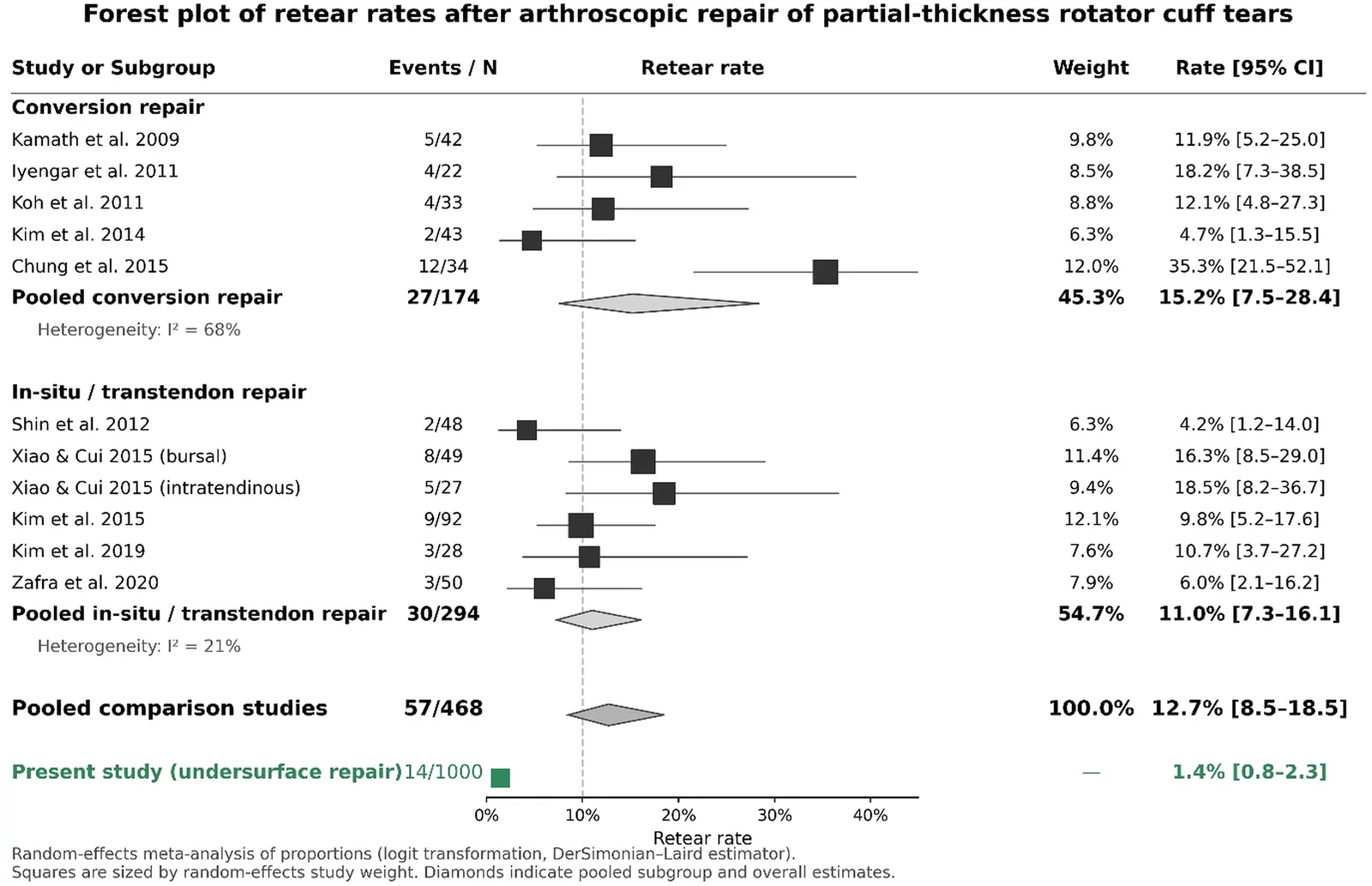
Forest plot of a pooled contextual summary of retear rates following arthroscopic repair of partial-thickness rotator cuff tears. *CI*, confidence interval.

A targeted literature search and a pooled summary of reported proportions were performed to provide context for the retear rates reported with alternative arthroscopic repair techniques. A targeted search of PubMed was performed using the following keywords: “partial thickness rotator cuff tear,” “arthroscopic repair,” “retear rate,” “structural integrity,” “conversion repair,” “transtendon repair,” “*in situ* repair,” and “biological augmentation.” The results are summarized in Figure 7.

### Conversion repair

Conversion repair involves completing the partial tear to a full-thickness defect prior to repair, typically performed via a bursal-sided approach with subacromial decompression. Kamath et al^18^ reported a 12% retear rate on ultrasound at a mean of 11 months in 42 patients. Iyengar et al^16^ found an 18% retear rate on MRI at a minimum of 2 years in 22 patients. Koh et al^23^ reported a 12% retear rate on MRI in 33 patients after full-layer bursal-sided repair. Kim et al^20^ (2014) found a 5% retear rate on MRI or ultrasound at a minimum of 12 months in 43 patients. Chung et al^6^ reported a retear rate of 35% in 34 patients and identified tendon quality as a significant prognostic factor for retear rate.

### In-situ and transtendon repair

*In-situ* and transtendon techniques preserve the intact portion of the tendon while repairing the delaminated portion. Shin et al^33^ reported a 4% retear rate on MRI at 6 months in 48 patients comparing transtendon and conversion techniques. Xiao and Cui reported retear rates of 16% in 49 patients with bursal-sided tears^38^ and 19% in 27 intratendinous tears^37^ using *in situ* repair. Kim et al^21^ (2015) compared *in situ* and conversion repairs in 92 patients and reported a 10% retear rate with no significant difference between techniques. Kim et al^22^ (2019) reported an 11% retear rate in 28 patients with intratendinous tears. Zafra et al^42^ found a 6% retear rate on MRI at 12 months in 50 patients in a prospective randomized comparison of single-row transtendon and double-row suture bridge techniques, with no significant difference in functional outcomes between groups.

### Biological augmentation

Biological augmentation with bioinductive collagen implants has been proposed as an adjunct or alternative to standard repair for partial-thickness tears. These constructs are placed over tendon defects to provide structural support and act as a scaffold for tendon regeneration. Marin et al^26^ investigated 93 patients treated with a bioinductive collagen patch at a minimum 2-year follow-up, including 85 patients with partial-thickness tears, 76 of whom underwent patch implantation without concomitant rotator cuff repair. Retear was reported in only 4 patients; however, 19 patients experienced post-operative stiffness requiring surgical treatment or persisting beyond 6 months. In a propensity-matched trial, Yeazell et al^40^ reported that partial-thickness repairs augmented with a bioinductive bovine collagen patch had significantly higher rates of post-operative stiffness and reoperation compared with repair alone.

### Pooled retear rates

Across the 11 published series identified, a total of 468 patients underwent arthroscopic repair of partial-thickness rotator cuff tears using a variety of techniques, with 57 retears reported. The combined retear rate across these series of alternative repair techniques was 13% (95% CI: 9-19%), with moderate heterogeneity between studies. When stratified by technique, the combined retear rate was 15% (95% CI: 8-28%) for conversion repair and 11% (95% CI: 7-16%) for *in situ*/transtendon repair. These figures were numerically higher than the 1.4% (95% CI: 0.8-2.3%) retear rate observed in the present cohort. This was a contextual pooled summary rather than a formal systematic review and is subject to selection bias and to heterogeneity in imaging modality, follow-up duration, and patient populations across studies; direct comparison is therefore not possible (Fig. 6).

## Discussion

This study showed that an arthroscopic undersurface approach to the supraspinatus was effective for repairing partial-thickness rotator cuff tears. Restoration of structure was effective in this cohort, with 99% of patients demonstrating an intact tendon on ultrasound at 6-month follow-up. No cases required reoperation for infection or post-operative stiffness.

The observed retear rate of 1.4% was numerically lower than those reported in many published series, although direct comparison is not possible because of substantial methodological heterogeneity in imaging modality, follow-up duration, tear morphology, and patient selection. Unlike transtendon or bursal-sided approaches, the undersurface technique allows direct visualization of the articular surface tear, enabling precise suture placement and optimized tendon-to-bone compression. This undersurface view where the torn tendon was visualized with the arthroscope positioned in the glenohumeral joint, provides an unobstructed view of both the tear, long head of the biceps, capsule, and labrum. Furthermore, by limiting dissection to the glenohumeral space and avoiding entry into the subacromial bursa, this technique minimizes disruption of the biological repair environment and maximizes the existing tendon. There are some studies that support the preservation or reimplantation of the subacromial bursal tissue to rotator cuff repairs where it serves as a source of mesenchymal stem cells and the production of inflammatory mediators to possibly encourage tendon healing.^11^^,^^27^

There are a variety of potential reasons to further explain a low retear rate. Our use of a tension band mattress suture technique may have contributed to more robust repairs by improving footprint contact pressure and reducing suture pull-through within the tendon.^1^ Partial-thickness tears generally have smaller dimensions and less tendon retraction than full-thickness tears, which could have allowed for lower-tension repairs.^35^^,^^36^ From a biological standpoint, partial-thickness tears exhibit stronger healing responses when compared to full-thickness tears and are characterized by increased fibroblast cellularity and increased angiogenesis.^5^^,^^28^ Our study found that larger tear areas were associated with reduced pain, which may reflect a relatively attenuated biological healing response, potentially characterized by reduced cytokine activity.^4^ Following surgery, range of motion improved significantly from pre-operative and 6-week levels at 6 months, alongside strength and pain scores.

This series had an excellent safety profile. No patient required additional surgery for infection or post-operative stiffness. Retears, the only clinically significant complication, occurred in 14 patients (1.4%) and were managed according to patient preference and tear characteristics: 7 underwent revision rotator cuff repair, 3 were managed non-operatively, and 4 were lost to follow-up. Of those who underwent revision repair, 2 ultimately required reverse total shoulder arthroplasty, as did one of those managed non-operatively. The low complication burden may be an advantage of this technique compared with bioinductive patch augmentation, where stiffness rates of up to 20% have been reported. A study by Marin et al^26^ investigated 93 patients with rotator cuff tears at a minimum follow-up of 2 years who underwent treatment with a bioinductive collagen patch. Eighty-five patients had partial thickness tears and 76 underwent patch implantation without concomitant rotator cuff repair. While retear was only reported in 4 patients, their study reported relatively high rates of post-operative stiffness, with 19 patients overall experiencing stiffness that required treatment or persisted beyond 6 months. A propensity-matched trial by Yeazell et al^40^ similarly demonstrated higher stiffness and reoperation rates in partial thickness repairs augmented with a bovine collagen patch compared with repair alone. However, comparisons with bioinductive patch augmentation should be interpreted cautiously: indications, patient selection, and outcome definitions differ between those series and ours, and bioinductive implants may have a role in tears or patient circumstances not represented in the present cohort.

There are several limitations to this study. Firstly, as a single-surgeon, single-center study, the findings may lack external validity and may not be generalizable to other clinical settings. The operating surgeon had extensive experience in shoulder arthroscopy. Our learning curve analysis included a descriptive moving average that was exploratory and was therefore not subjected to formal change-point modeling. Structural integrity was assessed at a minimum of 6 months. Although most retears in published series are detected within the first 3 to 6 months post-operatively, late structural failure can occur, and tendon remodeling and functional recovery may continue beyond this point. Our data therefore cannot establish the durability of the repair, and our results are not directly comparable with series reporting 2-to 5-year structural outcomes. Longer-term follow-up of this cohort is ongoing. Clinical outcomes were assessed with validated, reliability-tested measures of range of motion, strength, and pain and with individual function items derived from the L'Insalata, Constant, and American Shoulder and Elbow Surgeons (ASES) instruments, reported separately for transparency rather than as a single composite. Our study also lacked a control group for direct comparison with other operative techniques, and the comparisons made here rely on historical series with heterogeneous imaging modalities. The low retear rate in this study, however, suggests that equipoise and superiority would be difficult to achieve in a randomized clinical trial. No author is an employee of, or receives royalties from, the manufacturer of the devices used in this study. The manufacturer provided no funding and had no role in the design, conduct, analysis, or reporting of this study.

## Conclusion

The results of this study indicate that an arthroscopic undersurface knotless inverted mattress repair of partial-thickness rotator cuff tears was very successful in achieving repair integrity with negligible morbidity. The favorable structural outcomes with this technique may relate, in part, to minimal disruption of the remaining tendon and subacromial bursa.

## Disclaimers

Funding: The ultrasound machine was provided to our research group at a reduced price to conduct research.

Conflicts of interest: The authors, their immediate families, and any research foundations with which they are affiliated have not received any financial payments or other benefits from any commercial entity related to the subject of this article.
